# A1 BACTERIAL LYSOPHOSPHATIDYLCHOLINE (LPC) AND LYSOPHOSPHATIDIC ACID (LPA) INDUCE VISCERAL HYPERSENSITIVITY THROUGH TRANSIENT RECEPTOR POTENTIAL CANONICAL 5 (TRPC5) AND LYSOPHOSPHATIDIC ACID RECEPTOR 1 (LPAR_1_) AND 3 (LPAR_3_) DEPENDENT-MECHANISMS

**DOI:** 10.1093/jcag/gwae059.001

**Published:** 2025-02-10

**Authors:** J Pujo, G De Palma, J Lu, G H Rueda, F A Vicentini, M Hall-Bruce, A Nardelli, E Verdu, D E Reed, S J Vanner, S Collins, P Bercik

**Affiliations:** Gastroenterology, Farncombe Family Digestive Health Research Institute, Hamilton, ON, Canada; Gastroenterology, Farncombe Family Digestive Health Research Institute, Hamilton, ON, Canada; Gastroenterology, Farncombe Family Digestive Health Research Institute, Hamilton, ON, Canada; Gastroenterology, Farncombe Family Digestive Health Research Institute, Hamilton, ON, Canada; Gastroenterology, Farncombe Family Digestive Health Research Institute, Hamilton, ON, Canada; Gastroenterology, Farncombe Family Digestive Health Research Institute, Hamilton, ON, Canada; Gastroenterology, Farncombe Family Digestive Health Research Institute, Hamilton, ON, Canada; Gastroenterology, Farncombe Family Digestive Health Research Institute, Hamilton, ON, Canada; Queen’s University, Kingston, ON, Canada; Queen’s University, Kingston, ON, Canada; Gastroenterology, Farncombe Family Digestive Health Research Institute, Hamilton, ON, Canada; Gastroenterology, Farncombe Family Digestive Health Research Institute, Hamilton, ON, Canada

## Abstract

**Background:**

Abdominal pain is the key symptom in Irritable Bowel Syndrome (IBS). Its pathophysiology is not fully understood, but low-grade inflammation and gut microbiota-diet interactions have been implicated. LPC and LPA are phospholipids generated by inflammatory processes in mammals, which are known to induce neurogenic pain through multiple channels and G-protein-coupled receptors. It is unknown whether bacteria can produce LPC/LPA and through which pathways they signal to the host.

**Aims:**

To investigate whether bacterial LPC and LPA induce visceral hypersensitivity and identify the underlying mechanisms.

**Methods:**

Germ-free NIH Swiss and Swiss Webster mice (n=117) were colonized for 5 weeks with fecal microbiota from IBS patients with either low or high fecal LPC/LPA levels, or microbiota from healthy controls. The mice were fed either regular chow or a diet enriched with phosphatidylcholine (PC) (2g/kg) a precursor of LPC and LPA. Visceral sensitivity was assessed by visceromotor responses (VMRs) to colorectal distension. Dorsal root ganglion (DRG) neurons from C57BL/6 mice were pretreated with a TRPC5 inhibitor (AC1903; 30 μM), an LPAR_1_ antagonist (AM095; 10 μM), or an LPAR_1_ and LPAR_3_ antagonist (Ki16425; 10 μM) or their vehicle (0.1% DMSO), and then treated with LPC (10 μM) or LPA (10 μM), respectively. Calcium mobilization was measured by a Cytation C10 imaging reader using a fluorescent probe Fluo-4 (1 mM). C57BL/6 mice received intracolonic infusion with a vehicle or a mix of LPC (600 μM) /LPA (25 μM), one group of mice was pretreated with AC1903 (30 μM) and Ki16425 (10 μM) prior to LPC/LPA administration, CRD was performed 90 minutes later.

**Results:**

Mice with fecal microbiota from IBS patients with high fecal LPC/LPA displayed visceral hypersensitivity compared to mice with microbiota from healthy controls or from patients with low fecal LPC/LPA. These increased responses were seen only in mice fed PC-enriched diet, but not with regular chow. Both LPC and LPA administration increased the percentage of responding DRG neurons compared to the vehicle. The pretreatment with AC1903, AM095 or Ki16425 decreased the percentage of responding neurons when treated with LPC or LPA, respectively, compared to the vehicle. Intracolonic administration of LPC and LPA induced a significant increase in visceral sensitivity compared to the vehicle-treated mice. This increase was prevented in mice pretreated with an intracolonic injection of AC1903 and Ki16425.

**Conclusions:**

Bacterial LPC and LPA induce neuronal activation and visceral hypersensitivity through TRPC5 and LPAR_1_/LPAR_3_-dependent mechanisms, respectively.

**Funding Agencies:**

CIHRWeston Family Foundation

